# Genetic variation in the odorant receptors family 13 and the mhc loci influence mate selection in a multiple sclerosis dataset

**DOI:** 10.1186/1471-2164-11-626

**Published:** 2010-11-10

**Authors:** Pouya Khankhanian, Pierre-Antoine Gourraud, Stacy J Caillier, Adam Santaniello, Stephen L Hauser, Sergio E Baranzini, Jorge R Oksenberg

**Affiliations:** 1 Department of Neurology, University of California, San Francisco, CA 94143-0435, USA

## Abstract

**Background:**

When selecting mates, many vertebrate species seek partners with major histocompatibility complex (MHC) genes different from their own, presumably in response to selective pressure against inbreeding and towards MHC diversity. Attempts at replication of these genetic results in human studies, however, have reached conflicting conclusions.

**Results:**

Using a multi-analytical strategy, we report validated genome-wide relationships between genetic identity and human mate choice in 930 couples of European ancestry. We found significant similarity between spouses in the MHC at class I region in chromosome 6p21, and at the odorant receptor family 13 locus in chromosome 9. Conversely, there was significant dissimilarity in the MHC class II region, near the *HLA-DQA1 *and -*DQB1 *genes. We also found that genomic regions with significant similarity between spouses show excessive homozygosity in the general population (assessed in the HapMap CEU dataset). Conversely, loci that were significantly dissimilar among spouses were more likely to show excessive heterozygosity in the general population.

**Conclusions:**

This study highlights complex patterns of genomic identity among partners in unrelated couples, consistent with a multi-faceted role for genetic factors in mate choice behavior in human populations.

## Background

When selecting mates, individuals of many vertebrate species favor partners with heterologous major histocompatibility complex (MHC) genes [1-6]. These genes have immune-recognition and response functions, thus this behavior can be interpreted as a mechanism developed through evolution to prevent inbreeding and increase MHC diversity, imparting more robust immune systems to offspring [7-10]. However, translation of these observations to human mating is not straightforward and the dependence of human mate selection on genetic factors, including the MHC, remains controversial [11-18]. Individuals in some populations, European American couples from Utah [13,14] and Hutterites [19], have been found to favor MHC-dissimilar mates. Other populations show no strong evidence of MHC-selective mating, including Yorubans in Nigeria [13,14], South Amerindians [20], Dutch [21], Japanese [22], Swedish [23], Uruguayans [24], and Caucasians [25]. On the other hand, evidence for preference of MHC-similar mates was seen in Tohoku Japanese [22] but only when considering extended haplotypes composed of alleles of the HLA genes A, B, C, DR, and DQ in linkage disequilibrium. MHC similarity among mates was also seen in a multi-population study [26]. "Facial preference" studies generally show no preference for MHC similarity or dissimilarity, although a preference for heterozygosity could not be ruled out [27-31]. Early "sweaty t-shirt" studies suggested that MHC-dissimilarity mediates odor preference for a potential mate, but follow-up studies highlighted a variety of confounding factors in this phenomenon, such as genetic background, sex, and contraceptive use [31-36], providing a partial explanation for the conflicting results.

The recent availability of highly efficient genome-wide genotyping platforms affords deeper marker saturation in regions of interest as well as the performance of hypothesis-neutral screens. In this study, a screen with 309,100 single nucleotide polymorphisms (SNPs) in 930 unrelated couples of European ancestry was used to assess genetic similarity between spouses. Quality-controlled genotypic data was obtained from the International Multiple Sclerosis Genetics Consortium (IMSGC) from a study performed to identify multiple sclerosis (MS) susceptibility genes [37]. In a hypothesis-neutral approach, we looked for similarity at the genome-wide level, at the regional level, and at the individual SNP level. A Benjamini-Hochberg [38] tail-area-based correction for multiple comparisons was applied to strictly control for false positives. An excess of SNP-level similarity was observed in class I of the MHC, and in a locus on chromosome 9 near eight consecutive functional odorant receptor genes. The results are consistent with a significant but multifaceted role for genetic factors influencing mate selection in humans.

## Results

Genetic similarity/dissimilarity between spouses was assessed using three parallel approaches. Relatedness coefficient R1 (defined in methods) measures similarity across genetic regions, while R_2 _measures similarity at individual SNPs. A third approach seeks the preponderance extreme R_2 _values across regions. In this way, we allowed for a region to exhibit similarity and dissimilarity independently. This is important for gene-rich regions such as the MHC, which could potentially have an intricate role in mate preference. Relatedness coefficients are positive when spouses are more similar than random pairs of individuals, and negative when spouses are more dissimilar.

### Genome-Wide Similarity

Genome wide, the partners in the 930 couples of European descent included in this study are more genetically similar than expected by chance (R1 = 0.00152, positive values of R1 indicate genetic similarity; *p *< 10^-6^, using 10^6 ^permutations). In apparent contrast, Chaix et al. [13] reported no genetic similarity in the 28 spouses from the HapMap CEU population (R1 = -0.00016, *p *= 0.739). Is their population different from ours? To answer this question, similarity (R1) was measured in 100,000 randomly selected sets of 28 couples from our dataset. The random set of 28 couples had R1 lower (less similarity) than that observed in the Chaix et al. study in 7.06% of the permutations. Although the power to assess differences in genetic identity patterns between the two populations is limited, it appears that the two populations are different. Further, the Chaix et al. results may be influenced by a few outlying couples [14].

### Hypothesis-Neutral Results

Using sliding windows of 3.6 Mb (in 100 Kb increments), there were 21,665 regions with at least 300 SNPs. The top 100 regions exhibiting excessive similarity or dissimilarity (extreme values of R1) are shown in Additional file 1: Table S1. After correction for multiple-hypothesis testing, none of these regions remain statistically significant (fdr ≈ 1). Additional file 2: figure S1 shows a relationship between R1 and recombination rate similar to that found in figure 2 of Chaix et al. [13], with more extreme values of R1 seen in regions of lower recombination rates. Hence, an examination of R1 at the locus level yielded no significant results after correction for multiple comparisons.

**Figure 1:**
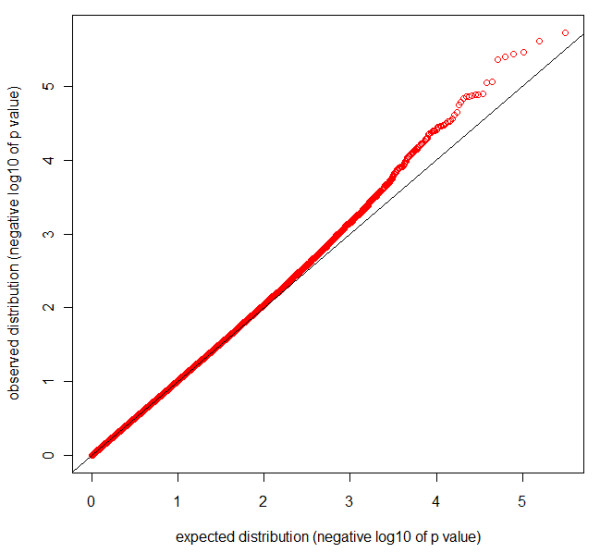
***P*-P plot of SNP level results**. *p *values of correlation among couples are plotted for all SNPs as a function of the normal distribution. The black line is equal to the expectation on H0. Overall, the data follows the normal distribution with an excess in the tail at *p *< 10^-4^.

On the other hand, using Pearson Correlation (R2) for SNPs that showed significant similarity or dissimilarity among couples in the screen, 38 individual SNPs passed the genome-wide significance threshold (fdr < 0.1) (Figure 1, Additional file 3: Table S2) after applying a Benjamini-Hochberg correction for multiple comparisons (n = 309,100 SNPs). A large proportion (33 of 38) of these SNPs exhibit spousal identity, in line with the genome-wide observations. Of these, 10 SNPs came from a region upstream from the 8 consecutive odorant receptor family 13 genes on chromosome 9.

An examination of the Fisher meta value at the regional level is shown in Additional file 4: Table S3. The top 100 regions exhibiting an abundance of significant SNPs with spousal identity are concentrated in two areas. The first is the odorant receptor (OR) 13 region on chromosome 9 (13-17.4 Mbp), and the other is on chromosome 11 (61.6-63.6 Mbp). The top 100 regions showing abundance of dissimilar SNPs (Additional file 5: Table S4) are also concentrated in two areas, chromosome 2 (13-17.4 Mbp) and chromosome 9 (36.9-38 Mbp). The abundance of similarity or dissimilarity found in these regions was not significant after correction for multiple comparisons.

### The MHC

Using R1, we observed an overall trend of similarity across the entire MHC that is not significant (R1 = 0.0051, uncorrected *p *= 0.076). When considering the results emerging from the MHC region, however, it is important to bear in mind the origin of our dataset: couples with an offspring affected with MS, an autoimmune disease with a genetic component, and a known strong association with the MHC, specifically with the relatively common HLA-DRB1*15:01 allele. Other genes within the HLA class I region have been also proposed to be independently linked to MS by conferring protection. In apparent contrast, Chaix et al reported significant dissimilarity in this region (R = -0.043, *p *= 0.015) for the 28 HapMap couples. Once again we ask whether their population was different from ours. When R1 was measured in 100,000 random sets of 28 couples chosen from our study, a value of R1 lower than -0.043 was only observed 0.853% of the time, suggesting significant differences between the two populations and reflecting the greater diversity of the dataset used in this study as well as the sampling variability of the HapMap samples [14].

Using R2, no individual SNP from the MHC passed the genome-wide threshold of significance. The top MHC SNP *rs2844731 *(R2 = 0.113, *p *= 0.00063, fdr = 0.42) was 291st on the list (sorted by significance) (Additional File 3: Table S2). The closest non-pseudo-gene is HLA-E.

**Figure 2:**
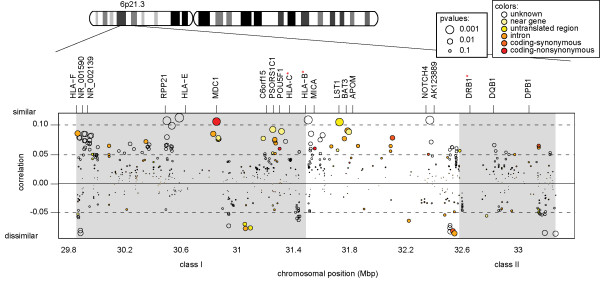
**MHC correlation plot**. The y-axis shows the correlation between couples at each SNP. Positive correlation means that the SNP shows identity between couples; negative correlation means that the SNP shows dissimilarity between couples. The size of the points is proportional to the SNP-wise significance. The color of the points indicates the function of the SNP (from UCSC). The MHC region is a mosaic of positive and negative correlations. Classes I and II are shaded grey. Proposed MS-related genes are highlighted with red stars. Positions and gene symbols are from UCSC (build 36).

Using the Fisher meta value, the MHC region as a whole showed a greater excess of significantly similar SNPs than the rest of the genome (Fisher meta value = 7.7 * 10^-10 ^, *p *= 0.013). Upon breaking down the MHC into three classes, significant genetic identity among couples was found in class I (Fisher meta value = 1.0 * 10^-8 ^, *p *= 0.029) (Table 1). Much of the similarity was due to a series of markers near *HLA-E *and *RPP21 *(a gene involved in maturation of rRNA), and to a lesser extent to another series of markers near *MDC1 *(a mediator of DNA damage checkpoint), including the non-synonymous SNP *rs9262152 *(Figure 2). We chose to perform PCR-based genotyping on this single SNP because of its relatively high correlation within the MHC (R2 = 0.106, *p *= 0.0012) and its non-synonymous nature. Observed similarity among couples at *rs9262152 *was confirmed by PCR (R2 = 0.091, *p *= 0.037, n = 387 couples) in a subset of the same couples but results were not replicated in independent couples (R2 = -0.0047, *p *= 0.5, n = 393), indicating that regional class I similarity is not a consequence of this SNP.

**Table 1:** Regional spousal identity.

Similarity only	SNPs	Fisher meta	Background	P value
		Value	Mean Fisher	
			Value	
HLA region	458	7.7 * 10^-10^	0.15	0.013
HLA class I region	280	1.0 * 10^-8^	0.043	0.029
HLA class II region	90	0.82	0.35	0.83
HLA class III region	88	6.4 * 10^-6^	0.36	0.09
OR13 region chr. 9	25	3.8 * 10^-43^	0.48	8.8 * 10^-5^

**Dissimilarity only**	**SNPs**	**Fisher meta**	**Background**	**P value**
		**Value**	**Mean Fisher**	
			**Value**	

HLA region	246	0.11	0.61	0.20
HLA class I region	140	0.43	0.56	0.39
HLA class II region	63	0.063	0.59	0.12
HLA class III region	43	0.24	0.60	0.27
OR13 region chr. 9	60	0.24	0.41	0.48

**Combined**	**SNPs**	**Fisher meta**	**Background**	**P value**
**Similarity and Dissimilarity**		**Value**	**Mean Fisher**	
			**Value**	

HLA region	704	7.5 * 10^-9^	0.51	0.0025
HLA class I region	420	7.3 * 10^-7^	0.033	0.23
HLA class II region	153	0.38	0.49	0.44
HLA class III region	131	2.6 * 10^-5^	0.53	0.047
OR13 region chr. 9	85	7.7 * 10^-29^	0.52	0.00056

**Position**	**Recombination rate cM/Mb**		**Start**	**End**

HLA region	1.57		29,700,000	33,300,000
HLA class I region	1.38		29,700,000	31,500,000
HLA class II region	2.43		32,500,000	33,300,000
HLA class III region	1.12		31,500,000	32,500,000
OR13 region chr. 9	0.69		104,000,000	104,500,000

The MHC region as a whole shows similarity between couples. When broken down into classes, class I shows significant similarity between couples. Of the three MHC classes, class II shows the most dissimilarity between couples, albeit not statistically significant. HLA-B and HLA-DRA denote the boundaries between the three MHC classes. UCSC Build 35 coordinates are shown.

Genetic similarity among couples was not ubiquitous throughout class I; a locus of dissimilarity was found at 31.0 Mbp, within a gene-rich region including *GTF2H4 *(general transcription factor involved in nucleotide excision repair), *SFTPG *(surfactant associated protein), *DPCR1 *(diffuse panbronchiolitis critical region) and *VARS *(valyltRNA synthetase 2). The identity pattern detected using Affymetrix arrays was confirmed by a dense Illumina custom array of 1536 MHC SNPs performed on 920 of the 930 couples (Additional file 6: Figure S2, Additional file 7: Table S5) [39].

Next, imputation of 6 classical HLA alleles (A, B, C, DRB1, DQA1, and DQB1) was performed using this dense coverage (Additional file 8: Table S6). There was significant dissimilarity in two of the three class II genes, *DQA1 *(*p *= 0.001) and *DQB1 *(*p *= 0.044).

### Functional Odorant Receptor Family 13

**Figure 3:**
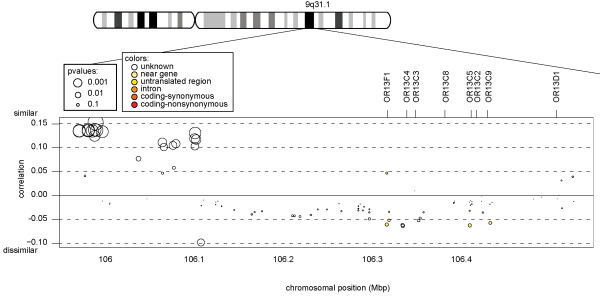
**OR13 correlation plot on chromosome 9**. A set of markers 200-300 Kbp upstream from 8 consecutive *OR13 *genes show excessive similarity between couples. Positions and gene symbols are from UCSC (build 36). The y-axis follows the convention of Figure 2.

Functional odorant receptor (OR) families (13 and 18) are located in clusters on various chromosomes. Only one such cluster (from family 13) was large enough for analysis. A 500 Kbp region covering 8 consecutive *OR13 *genes (*F1, C4, C3, C8, C5, C2, C9, and D1*) is bounded by *SMC2 *and *NIPSNAP3A*. This region exhibits the greatest abundance of SNP-level allelic similarity between couples in the entire genome (Fisher meta value = 3.85 * 10^-43 ^, *p *= 8.8 * 10^-5^). The bulk of genetic similarity is found in a non-coding region toward the centromere, 200-300 Kbp upstream from the OR13F gene cluster (Figure 3), even though there was adequate SNP coverage near the coding regions. The similarity in one SNP from this region (*rs1450686*) was confirmed by PCR-based genotyping in 387 couples from the original analysis (R2 = 0.17, *p *= 0.00056) and 393 new couples (R2 = 0.11, *p *= 0.016).

### Multiple-Sclerosis-Associated SNPs

Eleven non-MHC SNPs covered in this study are reportedly associated with MS risk. Similarity between couples (R2) is not significant at these SNPs, after a correction for 11 multiple hypotheses (Additional file 9: Table S7). Interestingly, 9 of 11 SNPs (including 2 that reach an uncorrected level of significance) show a trend for dissimilarity between spouses.

### Association with Observed Homozygosity in the General Population

If humans tend to select mates that are similar to self at certain genetic loci, then we would expect those loci would show excessive homozygosity in the population over time (Additional file 10: Figure S3). To test this hypothesis, we searched for excessive homozygosity (versus Hardy-Weinberg equilibrium) in unrelated individuals from the HapMap CEU dataset. For this analysis, we filtered out SNPs with minor allele frequency (MAF) less than 1%. We considered all markers showing significant similarity or dissimilarity between couples (*p *< 0.024) and significant deviation from Hardy-Weinberg equilibrium (*p *< 0.024) (Hardy-Weinberg disequilibrium *p *values calculated with the software Plink [40]). The two *p *value cutoffs were chosen because they combine by Fisher's method to yield *p *< 0.005. Markers showing similarity among couples in the screening dataset were 5 times more likely to show excessive homozygosity in the HapMap population than markers showing dissimilarity between couples (X^2 ^= 12.6021, df = 1, *p *< 0.004, chi-square test), further validating our observations (Table 2).

**Table 2:** Excessive homozygosity in the general population.

Observed (expected)	Excessive heterozygosity	Excessive homozygosity
Dissimilar among couples	16 (7.97)	20 (28.03)
Similar among couples	13 (21.03)	82 (73.97)

SNPs that are dissimilar among couples in the IMSGC dataset tend to show excessive heterozygosity in the HapMap CEU samples, while markers that are similar among IMSGC couples show excessive homozygosity in the HapMap samples (chi-square *p *< 0.004, expected values shown in parentheses). In all, 131 SNPs showed significant similarity or dissimilarity among IMSGC couples (*p *< 0.024) and significant deviation from Hardy-Weinberg equilibrium in the independent HapMap CEU dataset (*p *< 0.024).

## Discussion

We performed a genome-wide analysis of genomic identity in 930 White couples of European ancestry, and report (i) a significant similarity in couples' genotypes at MHC class I (ii) novel significant similarity among couples in SNPs linked to the *Odorant receptor family 13 *region on chromosome 9; (iii) 38 SNPs whose alleles showed significant correlation between couples (*q *value < 0.1), 10 of which are upstream from eight consecutive *OR13 *genes on chromosome 9. This, to our knowledge, is the first genome-wide study of its size with regard to human mate selection. We report a complex but statistically significant role for genetic similarity in mate choice, in particular the genes of the MHC and odorant receptors. In the MHC the interaction is different from class to class and from gene to gene, indicating that the disparity in the literature regarding the role of the MHC in mate choice may be resolved with inspection of smaller genetic windows.

### MHC, Mate Selection, and Multiple Sclerosis

In mice, MHC genotypes have been shown to be a determining factor in mate selection in some strains but not in others [6]. Although more strains of mice prefer the scent of MHC-dissimilar individuals when selecting mates [41], or rather the scent of mice with MHC dissimilar to parental MHC [5], it has been shown that they prefer the scent of MHC-similar mice when selecting a nesting partner [42]. Humans overwhelmingly tend to pick one person as both mate and nesting partner; it is difficult then to resolve the two. Difficulty in extrapolation across species is exacerbated by the differences in reproductive strategies of mice (who tend to have more pregnancies per lifetime, with several pups per pregnancy) and humans (few pregnancies per lifetime, usually one child per pregnancy). Another difference is exposed when the proposed mechanism for murine MHC detection in mate selection is examined. Rodent mate selection is grounded in odorant sensations [43]. Specifically, mice respond to the scent of urinary proteins, which are naturally abundant in mice but mainly indicative of disease in humans [44-46].

Our results contribute to this ongoing exploration by finding an abundance of genetic similarity among couples (parents of children with MS) across class I of the MHC. Within class I, much of the similarity occurs near the HLA-E gene. HLA-E is a member of the non-classical class I genes (Ib), with characteristically low polymorphism across primates. Assortative mating may be driving the relatively low polymorphism in this gene. One of the drivers of high polymorphism in HLA genes is "crossing over" events during meiosis. Crossing over fails to increase polymorphism in homozygous individuals. Homozygous individuals are more frequent in the population when mating is associative.

This study sampled much older couples than the majority of previous studies. For 73% of the couples in this study, the mating event (successful breeding) occurred in the 1950's and 1960's. Just as we recognize that our findings of MHC class I similarity may be specific to the ethnicity of our population, we must allow that the results may be specific to this post-war generation.

When considering these results in the context of human mate selection, it is important to note that MS is a complex genetic disease strongly associated with the MHC class II gene *HLA-DRB1*. Our dataset is thus enriched for the set of common risk alleles of this gene (DRB1*15:01, and to a lesser extent DRB1*03:01) potentially leading to biased observations, but we report significant dissimilarity at this locus. While *HLA-DRB1 *confers the greatest susceptibility to MS, it has been proposed that other HLA genes, in this case conferring resistance (HLA-C [47] and HLA-B [39]), may exist in the MHC class I region. Interestingly, we observed primarily similarity in this locus. Altogether, the observed patterns of identity in the parents of the affected individuals appear to conflict with what is expected in an MS dataset; we assume that risk genes would show similarity among parents. This assumption is subject to debate, as the mechanisms of MHC-mediated genetic risk to MS are not well understood. Furthermore, it is important to note that MHC class I similarity in parents could conceivably lead to viral susceptibility in offspring, and that the Epstein-Barr virus has been linked to MS [48]. Similar studies in other trio format datasets from other diseases with MHC etiology will be of great value in testing the hypothesis that parents of disease individuals display a different pattern of MHC identity than parents of healthy children. Overall, the mosaic-like statistically significant pattern of association between MHC and mate choice at the class level (and also at the gene level) is remarkably complex, linked perhaps to the extreme functional diversity across the MHC and abundance of hot-spots and warm-spots of genetic recombination distributed differently among individuals [49]. This complexity may underlie the conflicting reports of genetic identity in the literature focused on a single or limited number of variants.

The consequences of any deviation from random mating for human disease, particularly autoimmunity, are unknown, but regions of extreme similarity or dissimilarity among parents of affected individuals may be related to the presence of susceptibility and/or resistance loci. Furthermore, random mating is a commonly accepted assumption for most statistical genetic models usually performed to assess genetic association. If non-random mating exists, association studies should correct for the expected departure from Hardy Weinberg equilibrium assumptions.

### Olfaction

Olfaction, the proposed mechanism for MHC recognition, is involved in a variety of mating-related behaviors. While there is no genetic evidence to directly link odorant receptors to mate selection, the connection between olfaction and sexual behavior is well established. Olfaction is a necessary step in the mating mechanism for rodents and the kin-recognition mechanism in both humans and rodents. Specifically, mice respond to the scent of naturally abundant urinary proteins [44-46]. Odorant bulb removal eliminates mating behavior in male mice and hamsters, while eliminating maternal behavior in female mice [43,50,51]. For humans, body odor detection is a mechanism for kin recognition and mate preference, especially for females [32,33,52,53]. Human body odor influences general mood, attention state, and females' proclivity towards males [54-57]. Although most are non-functional, odorant receptors (OR) represent the largest family of genes in the human genome. There are two human OR families, 13 and 18, with known ligands (having a variety of perceived odors including sweat) [58]. It remains unclear whether the genetic similarity observed between mates leads to sexual attraction through olfaction or simply implies similarities in odor recognition and food and other smell preferences that would be practical concerns for human couples who live and eat together.

The genetic identity in the region was found 200-300 Kb upstream from the cluster of 8 consecutive OR13 genes. This would indicate that it is not protein identity, but rather some form of long-range gene regulation that is associated with mate selection. Long-range enhancers found in gene deserts are known to act at distances of hundreds of kilo base pairs [59-64]. The bulk of genetic similarity is 10-100 Kbp downstream from *SMC2*.

## Conclusions

We have seen a complex role for genetic similarity in mate choice, in particular genes of the MHC and odorant receptor family 13. Regarding the MHC, the observed interaction is not ubiquitous throughout the 3.6 Mbp region. Rather, the interaction is different from class to class, and from gene to gene. As higher resolution scans and sample populations of other ancestries, environments, and phenotypes (in particular non-MHC diseases) become available, deeper analysis of the roles of individual genes and functional pathways in mate selection and implications for health and disease will become possible.

## Methods

### Datasets

a) 334,923 Single Nucleotide Polymorphism (SNPs) from the International Multiple Sclerosis Genetics Consortium (IMSGC) dataset, typed by Affymetrix 500K GeneChip in 931 European and European American couples (1862 individuals). Samples came from collections the University of California, San Francisco (417 couples), the Cambridge University Hospital Multiple Sclerosis Center (453 couples), and the Brigham and Women's Hospital (61 couples). The mean age was 71.2 (sd = 9.5) for males and 68.6 (sd = 9.2) for females. For 73% of couples, the mating event (successful breeding) occurred in the 1950's and 1960s. Divorce status and length of marriage was not recorded. One couple was removed because one parent was affected with multiple sclerosis (MS). 930 couples, each with a child afflicted with MS were used for analysis presented here. We included only autosomal SNPs with minor allele frequency above 5%, missing no more than 10% of genotypes in males or females (309,100 SNPs). This dataset is available on dbGAP (phs000139.v1.p1) [37].

b) 1,536 SNPs in the extended MHC region from the International MHC and Autoimmunity Genetics Network (IMAGEN) [39] dataset, typed by lllumina chip in 920 of 930 IMSGC couples. 1,078 SNPs passed quality control, 94 of which were in common between the two datasets.

c) A set of HLA types for 6 of the main HLA genes imputed from the IMAGEN SNP genotypes by the original authors.

d) Candidate SNPs were genotyped by PCR in 387 couples from the IMSGC dataset and 393 new European American couples. Both datasets were collected using the same inclusion criteria and have a similar distribution of age and ethnicity. Notably, these 393 new couples are also parents of children with MS.

### Relatedness Analysis

The following approaches for measuring genetic similarity were used in parallel.

a) In the IMSGC dataset, a relatedness coefficient R1 was defined for each couple at each variant as a ratio of probabilities of identity in state R1 = (Qc -Qm)/(1 -Qm), where Qc is the proportion of identical variants between the two spouses and Qm is the mean proportion of identical variants in the sample (an average over all possible pairs). For SNPs, the proportion of identical variants between two individuals is 1 if both individuals are homozygous for the same allele, 0.5 if either individual is heterozygous, and 0 otherwise. For larger genetic regions of at least 300 SNPs, Qc and Qm were averaged across all SNPs in the region. The significance of R1 was assessed using a permutation approach: the two-sided *p *value is the proportion of permutations (where spouses are shuffled) in which the mean R1 of permuted couples is more extreme than the mean R1 of real couples. 100,000 permutations were performed. This approach was used for HLA types and regions of SNPs by Chaix et. al. [13] and the relatedness coefficient is discussed by Rousset [65].

b) In the IMSGC dataset, another relatedness measure R2 was defined at individual SNPs across all couples as the Pearson correlation between fathers' and mothers' genotypes (recoded as 0, 1, and 2). A comparison of R2 and R1 at individual SNPs is discussed in Additional file 11: Text S1. Significance was assessed using two approaches, one based on permutation and another based on the genome as a background (Additional file 12: Text S2). Both methods yield similar results; we present the latter method.

c) In the IMSGC dataset, we next tested the hypothesis that an abundance of significant positive similarity exists among SNPs in a given region, say the MHC. For that purpose we combined the *p *values of all SNPs in the region which were similar between couples (R2 > 0 from part b) using Fisher's method [66]. A low Fisher meta value indicates a more significant finding. The Fisher meta value of the given region was contrasted against all other equally sized non-centromeric regions (looking only at SNPs exhibiting similarity, R2 > 0) from the entire genome (excluding the chromosome containing the candidate region) with a lower recombination rate than the candidate region if the candidate region has lower than average recombination rate (or a higher recombination rate than the candidate region if the candidate region has a higher than average recombination rate). The *p *value is assigned to each candidate region as the percent of regions across the genome that had Fisher meta values smaller than the Fisher meta value of the candidate region. In the hypothesis-neutral approach (where all regions genome-wide are considered), we apply a Benjamini Hochberg correction for multiple comparisons.

We also tested the hypothesis that an abundance of significant negative similarity exists among SNPs in a given region. For this, we repeated the above steps using only SNPs showing dissimilarity (R2 < 0). In this way, we allowed for a region to exhibit similarity and dissimilarity independently. This is important for gene-rich regions such as the MHC, which could potentially have a multifaceted role in mate preference.

d) Validation of observations in the MHC region was performed using the IMAGEN study. For each SNP, significance of the similarity score was assessed by 50,000 permutations. Regional scoring of the 3 MHC classes (Fisher meta value) was done in the same manner as the IMSGC. However, the procedure for assigning significance to the Fisher meta value was necessarily different from that used for the IMSGC dataset; with the IMAGEN dataset, we did not have the entire genome to use as a background. Instead, we created a regional background by shuffling couples 50,000 times, each time calculating the Fisher meta value on each of the 3 MHC classes. The percent of random Fisher meta values from the background that are lower than the observed Fisher meta value of each region is reported as the *p *value of that region.

e) For each of the six HLA genes from the IMAGEN study, a similarity score was calculated as follows. Each gene was given one point for each couple that shares one common allele at that gene, and two points for each couple that has both alleles in common. The similarity score for each gene was the total number of points across 920 couples. Couples were reassigned 20,000 times and similarity scores recalculated, creating a background distribution. *P *values were assigned using a cumulative normal distribution, with mean and standard deviation assessed from the background. Normality of the background distributions was assessed by visual inspection and the Anscombe-Glynn test of kurtosis.

f) For SNPs genotyped by PCR, significance of the similarity measure (Pearson correlation) was assessed by using a normal distribution with mean and standard deviation estimated from 200,000 random measurements (where the spouses were randomly re-assigned). Normality of the background distributions was assessed by visual inspection and the Anscombe-Glynn test of kurtosis.

### Linkage Disequilibrium

We checked that results are not affected by varying SNP density and linkage pattern across the genome by re-doing our analyses on reduced sets of approximately independent SNPs. Haplotype block tagging SNPs were selected genome-wide at r^2 ^thresholds of 0.25, 0.5, and 0.75 using software Plink.

### Control for Ethnic Diversity

An earlier study with a large sample size (n = 1,017 couples) that also found HLA similarity between couples suggested a possible confounding issue. The existence of ancestral or ethnic stratification with characteristic HLA types may influence the degree of genetic identity between couples [26]. Ancestry-related mate selection would appear as HLA-related selection because HLA is an excellent ancestry marker [67]. In our study, this issue is addressed foremost by comparing each candidate region or SNP to the entire genome. As a second layer of control, genome-wide pair-wise IBD distances (calculated with software Plink [40]) were used to cluster patients (using Ward agglomeration via the hclust function in R [68]) (Additional file 13: Figure S4). Outlying clusters of Mediterranean, Hispanic, Ashkenazi, and Eastern European couples were removed. All analysis was repeated on this smaller dataset of 803 couples. Results were largely unchanged.

In this paper, we chose to present the results from all 930 couples. When filtering for a more homogeneous western European population, we are removing a number of inter-group spouses (i.e. where one spouse is western European and the other is not). Inter-group mating events are a real phenomenon that we want to capture in the analysis.

### PCR

PCR validations and replications were done with a made-to-order Applied Biosystems TaqMan SNP genotyping assay, and carried out in 384-well plates using Applied Biosystems TaqMan genotyping Master Mix on an ABI Prism 7900HT Sequence Detection System using SDS 2.1 software.

### Imputation of HLA Alleles

Imputation of HLA Alleles from dense SNP coverage was performed by the authors of the IMAGEN paper [39]. HLA genotypes (at 2 digit resolution) were imputed from SNPs in the MHC using a recently developed approach [69]. The training database was from a previously created map of 7,500 SNPs, deletion insertion polymorphisms, and HLA alleles for 182 Utah residents (29 extended families containing 45 unrelated parent-offspring trios) of European ancestry in the Centre d'Etude du Polymorphisme Humain collection [70]. Up to 40 SNPs were used to impute each HLA allele. Note the significant overlap between the training dataset used here to impute HLA types and the dataset used by Chaix et al to assess HLA similarity between spouses.

## Authors' contributions

JRO, SLH, SEB, PAG, and PK conceived and designed the study. SJC carried out the rt-PCR assays. AS recoded HLA types and provided data management. PK performed the statistical analysis. JRO, SEB, and PK wrote the manuscript. All authors read and approved the final manuscript.

## Supplementary Material

Additional file 1 **Table S1. Top similarities at the regional level using R1**. Each of the top regions was compared against all regions from the genome with lower recombination rate if the region is lower than average, or higher recombination rate if the region is higher than average. On the far right, we see what results would look like using the more homogeneous subset (803 couples of Western European descent) of the population (see Methods). Click here for file

Additional file 2 **Figure S1. Average relatedness coefficient R1 between spouses at 3.6 Mb regions throughout the genome versus recombination rate**. More extreme values of R1 are seen in regions of lower recombination rates. The HLA region is shown in red. Compare to Figure 2 of Chaix et al. Click here for file

Additional file 3 **Table S2. Genome-wide SNP-level results**. Approximately 4,000 most highly correlated SNPs among the 930 IMSGC couples. This includes positive and negative correlation. All SNPs with a one-tailed *p *value of 0.01 or better are highlighted by filter. Click here for file

Additional file 4 **Table S3. Top similarities at the regional level using Fisher values**. Regions exhibiting an abundance of significantly similar SNPs (R2 > 0). Click here for file

Additional file 5 **Table S4. Top dissimilarities at the regional level using Fisher values**. Regions exhibiting an abundance of significantly dissimilar SNPs (R2 < 0). Click here for file

Additional file 6 **Figure S2. Validation on MHC results with the IMAGEN dataset**. In the screening IMSGC dataset, the MHC region (663 SNPs) was identified in the candidate-region approach as a mosaic of similarity and dissimilarity. 920 of the 930 couples were re-genotyped by a dense custom Illumina Platform (IMAGEN dataset: 1,078 SNPs passed quality control). (A) The pattern of similarity found in the IMAGEN dataset is comparable to that found in the screening (Figure 2). (B) 150 MHC SNPs were in common between IMAGEN and IMSGC. For each SNP passing quality control (94 SNPs), similarity between couples was calculated separately in both datasets. The correspondence of similarity scores between the two datasets was high (r^2 ^= 0.94). Click here for file

Additional file 7 **Table S5. IMAGEN regional p values**. Regional scoring of the 3 MHC classes (Fisher meta value) was done in the same manner as the IMSGC. P values are obtained by shuffling couples 50,000 times. Click here for file

Additional file 8 **Table S6. Imputed classical HLA alleles**. Two of the class II genes, *DQA1 *and *DQB1 *showed significant dissimilarity between couples. Two-digit allele designations were used. Click here for file

Additional file 9 **Table S7. Multiple-Sclerosis-associated SNPs**. Spousal identity (R2) and uncorrected p-value for 11 SNPs associated with multiple sclerosis. After correction for 11 multiple comparisons, the spousal identity is not statistically significant. Click here for file

Additional file 10 **Figure S3. Parental similarity versus offspring heterozygosity**. When parents choose mates that are similar to self at a given SNP, the result is excessive homozygosity in the children (an excess of homozygous genotypes at that SNP). Conversely, when parents choose mates that are dissimilar to self, the result is excessive heterozygosity in the children. In a simulation, random genotypes for 22,500 SNPs (2,500 with each MAF ϵ(0.05, 0.1, 0.15, 0.2, 0.25, 0.3, 0.35, 0.4, 0.45)) were generated for 1,000 sets of parents. At each SNP, the similarity measure (Pearson correlation) was calculated between the vectors of parental genotypes (shown on the y-axis). For each SNP, the genotypic frequencies of the offspring of the 1,000 sets of parents were calculated based on Mendelian inheritance. The observed frequency of heterozygotes in the offspring was divided by the expected frequency of heterozygotes, assuming Hardy Weinberg equilibrium (x-axis). A value higher than 1 on the x-axis means that offspring have a greater than expected frequency of heterozygotes, while a value smaller than 1 on the x-axis means that offspring display excessive homozygosity. These plots show that SNPs which show similarity between parents (high values on the y-axis) are more likely to show excessive homozygosity in the offspring (low values on the x-axis). To extend the concept: if parents select mates that are similar to self at a given SNP, over many generations we expect excessive homozygosity in the general population compared to Hardy Weinberg equilibrium. Click here for file

Additional file 11 **Text S1. Comparison of two measures of similarity**. Click here for file

Additional file 12 **Text S2. Comparison of two methods of assessing significance of Pearson Correlation as a measure of similarity**. Click here for file

Additional file 13 **Figure S4. Hierarchical clustering**. Using IBD distances calculated in software Plink, Ward agglomerative clustering (done in R) reveals a large cluster (A) of Scandinavian and western Europeans on the left. Smaller clusters on the right include (B) Eastern European (Russian and Polish) Ashkenazi Jews, (C) Mediterranean/Western European, (D) Hispanic with some Mediterranean, (E) Mediterranean, (F) non-Ashkenazi Eastern European. Self-reported ethnicity data was available for about 1/3 of the samples. This data is shown below the clusters. A red dot on the "Polish" row means that the person reports being Polish. A black dot means that the person did not report being Polish. The grey background means that no self-reported data was available for that person. Just above the self-reported ethnicity rows (black and red) is a single row showing cohort. Each sample belonged to one of three cohorts (UCSF = green, BWH = black, CMS = red). Note that nearly all samples from the non-western European group (B-F) came from the UCSF cohort. Click here for file
