# Supplementary figures and images for: Genetic variation in the odorant receptors family 13 and the mhc loci influence mate selection in a multiple sclerosis dataset
Source: BMC Genomics. 2010 Nov 10;11:626. doi: 10.1186/1471-2164-11-626 (PMC3091764; doi:10.1186/1471-2164-11-626)

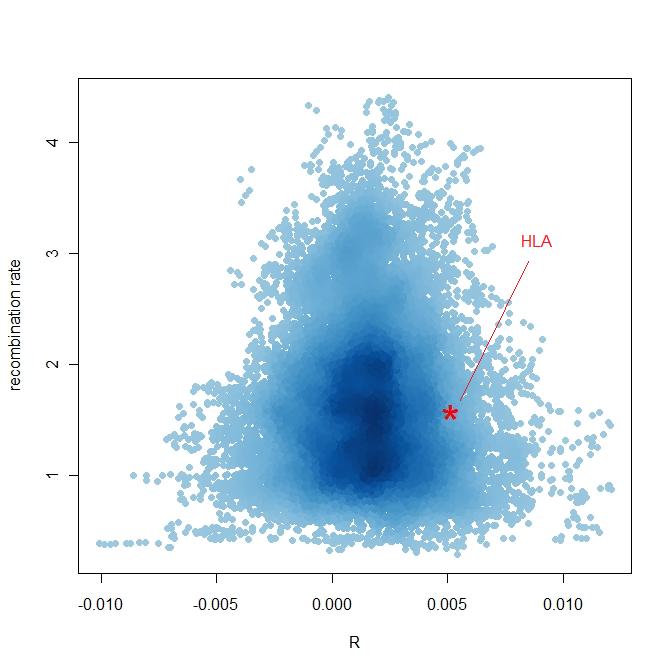

Supplement: Additional file 2 — Figure S1. Average relatedness coefficient R1 between spouses at 3.6 Mb regions throughout the genome versus recombination rate. More extreme values of R1 are seen in regions of lower recombination rates. The HLA region is shown in red. Compare to Figure 2 of Chaix et al. [file 1471-2164-11-626-S2.JPEG]

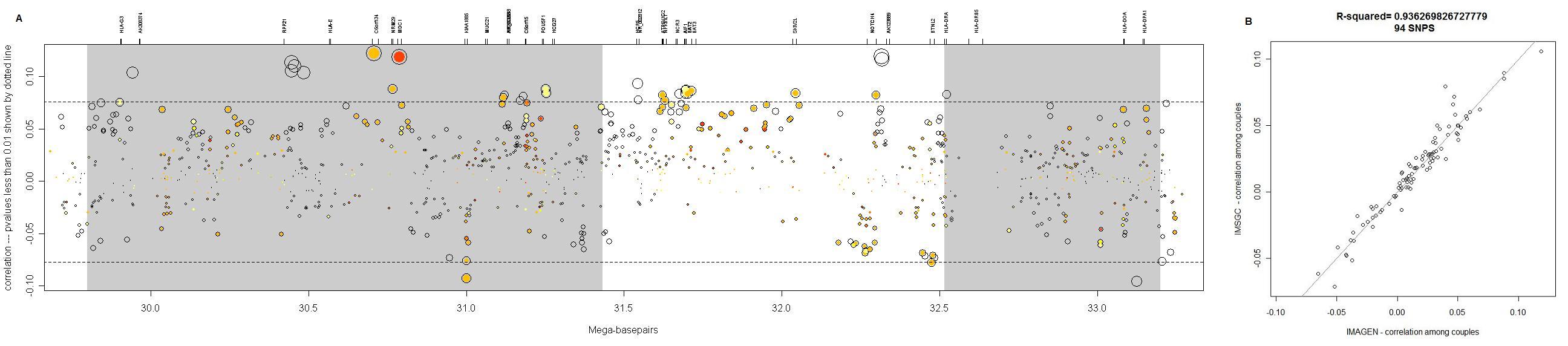

Supplement: Additional file 6 — Figure S2. Validation on MHC results with the IMAGEN dataset. In the screening IMSGC dataset, the MHC region (663 SNPs) was identified in the candidate-region approach as a mosaic of similarity and dissimilarity. 920 of the 930 couples were re-genotyped by a dense custom Illumina Platform (IMAGEN dataset: 1,078 SNPs passed quality control). (A) The pattern of similarity found in the IMAGEN dataset is comparable to that found in the screening (Figure 2). (B) 150 MHC SNPs were in common between IMAGEN and IMSGC. For each SNP passing quality control (94 SNPs), similarity between couples was calculated separately in both datasets. The correspondence of similarity scores between the two datasets was high (r2 = 0.94). [file 1471-2164-11-626-S6.JPEG]

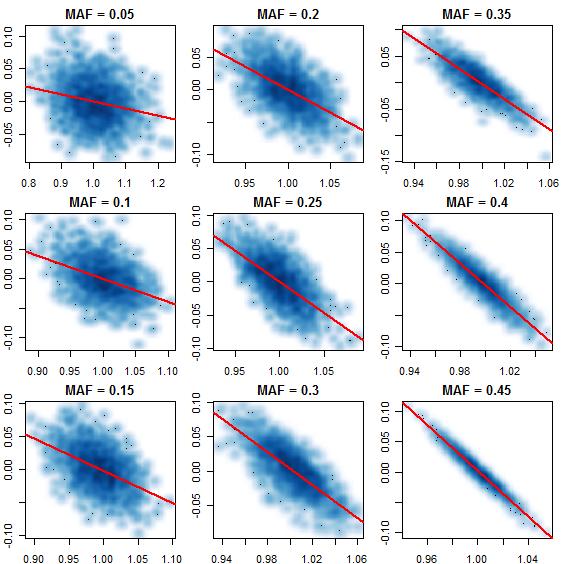

Supplement: Additional file 10 — Figure S3. Parental similarity versus offspring heterozygosity. When parents choose mates that are similar to self at a given SNP, the result is excessive homozygosity in the children (an excess of homozygous genotypes at that SNP). Conversely, when parents choose mates that are dissimilar to self, the result is excessive heterozygosity in the children. In a simulation, random genotypes for 22,500 SNPs (2,500 with each MAF ϵ(0.05, 0.1, 0.15, 0.2, 0.25, 0.3, 0.35, 0.4, 0.45)) were generated for 1,000 sets of parents. At each SNP, the similarity measure (Pearson correlation) was calculated between the vectors of parental genotypes (shown on the y-axis). For each SNP, the genotypic frequencies of the offspring of the 1,000 sets of parents were calculated based on Mendelian inheritance. The observed frequency of heterozygotes in the offspring was divided by the expected frequency of heterozygotes, assuming Hardy Weinberg equilibrium (x-axis). A value higher than 1 on the x-axis means that offspring have a greater than expected frequency of heterozygotes, while a value smaller than 1 on the x-axis means that offspring display excessive homozygosity. These plots show that SNPs which show similarity between parents (high values on the y-axis) are more likely to show excessive homozygosity in the offspring (low values on the x-axis). To extend the concept: if parents select mates that are similar to self at a given SNP, over many generations we expect excessive homozygosity in the general population compared to Hardy Weinberg equilibrium. [file 1471-2164-11-626-S10.JPEG]

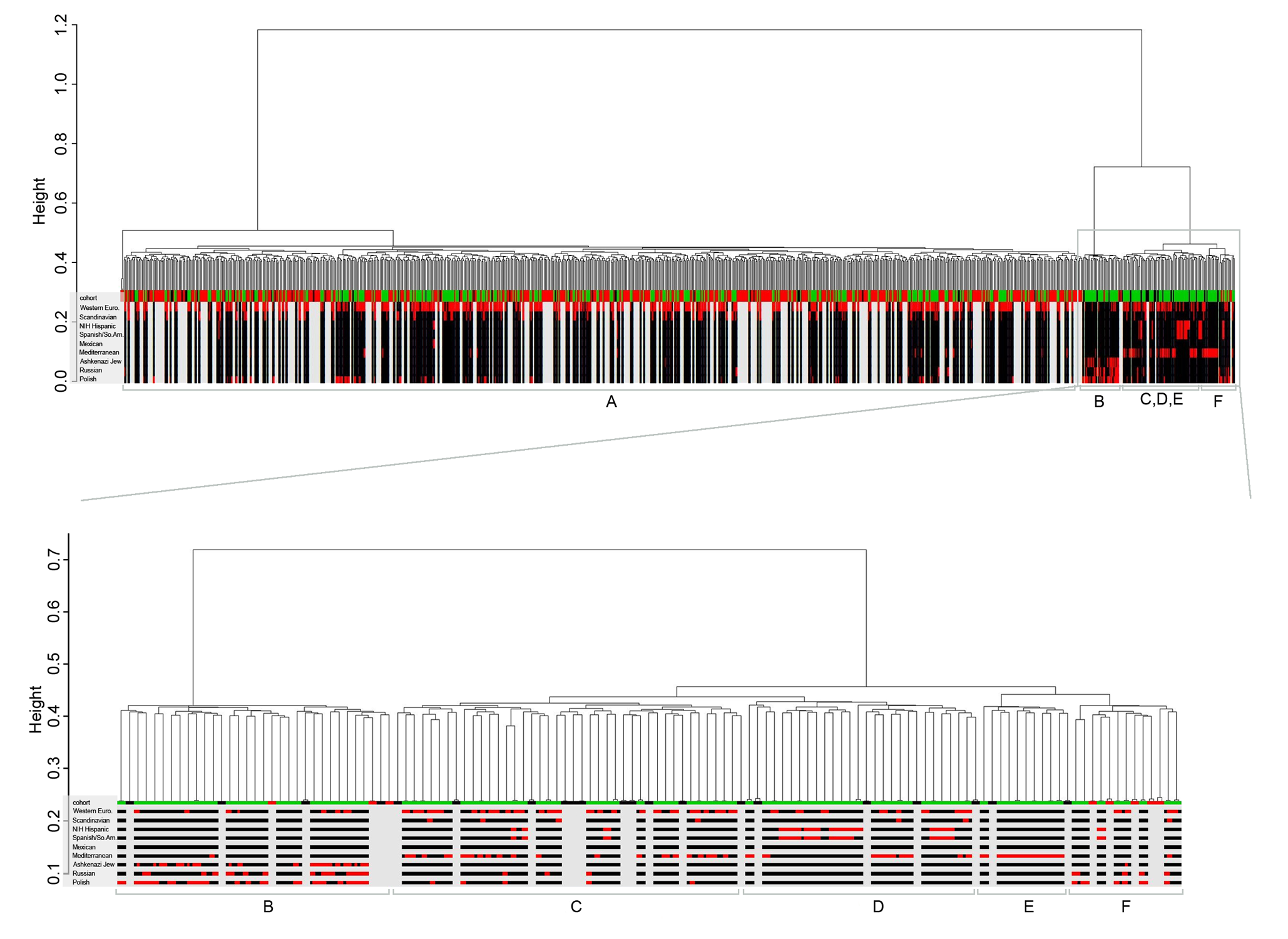

Supplement: Additional file 13 — Figure S4. Hierarchical clustering. Using IBD distances calculated in software Plink, Ward agglomerative clustering (done in R) reveals a large cluster (A) of Scandinavian and western Europeans on the left. Smaller clusters on the right include (B) Eastern European (Russian and Polish) Ashkenazi Jews, (C) Mediterranean/Western European, (D) Hispanic with some Mediterranean, (E) Mediterranean, (F) non-Ashkenazi Eastern European. Self-reported ethnicity data was available for about 1/3 of the samples. This data is shown below the clusters. A red dot on the "Polish" row means that the person reports being Polish. A black dot means that the person did not report being Polish. The grey background means that no self-reported data was available for that person. Just above the self-reported ethnicity rows (black and red) is a single row showing cohort. Each sample belonged to one of three cohorts (UCSF = green, BWH = black, CMS = red). Note that nearly all samples from the non-western European group (B-F) came from the UCSF cohort. [file 1471-2164-11-626-S13.TIFF]
