# Supplementary material for: Genetic variation in the odorant receptors family 13 and the mhc loci influence mate selection in a multiple sclerosis dataset
Source: BMC Genomics. 2010 Nov 10;11:626. doi: 10.1186/1471-2164-11-626 (PMC3091764; doi:10.1186/1471-2164-11-626)
Supplement: Additional file 8 — Table S6. Imputed classical HLA alleles. Two of the class II genes, DQA1 and DQB1 showed significant dissimilarity between couples. Two-digit allele designations were used. [file 1471-2164-11-626-S8.DOC]

Supplementary Table 6

| ***MHC Gene*** | ***Number of alleles*** | ***Similarity score*** | ***Random similarity scores*** | | ***One-sided p value*** |
| --- | --- | --- | --- | --- | --- |
|  |  |  | ***mean*** | ***st dev.*** |  |
| DRB1 | 23 | 359 | 367.4649 | 14.56368 | 0.280542 |
| DQA1 | 7 | 530 | 583.5012 | 17.25174 | 0.000964 |
| DQB1 | 14 | 481 | 508.7543 | 16.29615 | 0.044273 |
| A | 17 | 441 | 438.9042 | 15.75389 | 0.447083 |
| C | 17 | 337 | 328.8336 | 14.84049 | 0.291064 |
| B | 29 | 276 | 265.7033 | 13.3702 | 0.220613 |
