# Supplementary material for: Genetic variation in the odorant receptors family 13 and the mhc loci influence mate selection in a multiple sclerosis dataset
Source: BMC Genomics. 2010 Nov 10;11:626. doi: 10.1186/1471-2164-11-626 (PMC3091764; doi:10.1186/1471-2164-11-626)

# Supplementary methods -- Assessing Significance of the Pearson Correlation at Individual SNPs.

Similarity between couples was measured at individual SNPs. For each SNP genotypes were re-coded as 0, 1, and 2. The Pearson correlation between the male and female genotypes was computed as a measure of genetic similarity between couples. Genetic similarity measures were converted into two measures of significance, the first based on permutation and the second based on the genome as background. Both methods yield similar results. In this paper we report the second, more conservative, *p* value.

For each SNP, we created a random background by shuffling the genotype vectors (breaking the relationship between the couples) 5,000 times. The random backgrounds of SNPs with similar minor allele frequency (MAF) and similar heterozygosity (H) were pooled to create larger backgrounds. The MAF, which ranges from 0 to 0.5, was broken down into 25 equally spaced bins of size 0.02. The H (the observed heterozygosity calculated by plink) which ranges from 0 to about 0.55 was broken up into 28 equally spaced bins of size 0.02. Permutation *p* values were assigned using a cumulative normal distribution with mean and standard deviation assessed from the background.

We also implemented an independent strategy to measure statistical significance. The list of similarity measures of all SNPs was imported into fdrtool, a package in R which calculates *p* value and *q* value from Pearson correlation using genome distribution as a background [35]. The *q* value is a Benjamini Hochberg false discovery rate. The genomic distribution as a background is normally distributed (Anscombe-Glynn kurtosis test, kurtosis = 3.0013, *p* = 0.88, null hypothesis: kurtosis = 3, alternate hypothesis: kurtosis <> 3)*.*

All *p* values were one-tailed. *p* values obtained by permutation are similar to the *p* values obtained using the genome as background via the R-package *fdrtool* (Supplementary text figure). Negative log10 of *p* values obtained by using the genome as a background (y-axis) are comparable to *p* values from permutation (x-axis). All points lie below the identity line, indicating use of the genome as background is the more conservative approach. In this paper, we report *p* values obtained using the genome as a background using *fdrtool*.


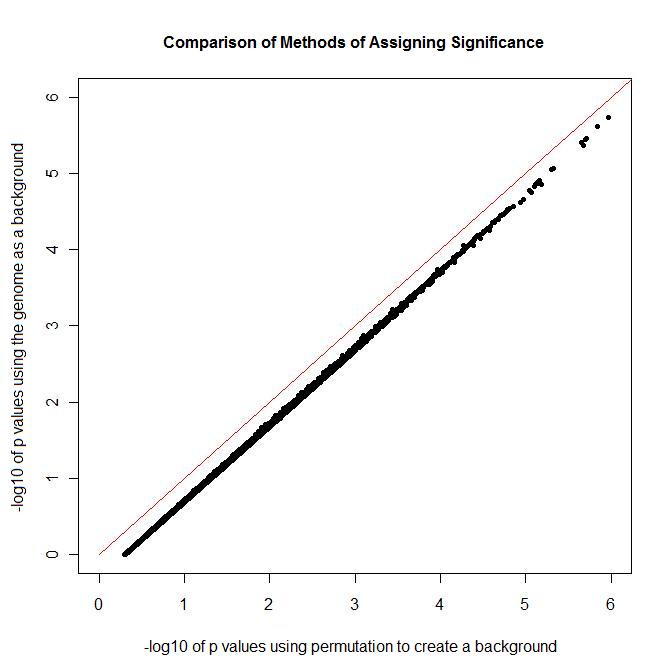

Supplement: Additional file 11 — Text S1. Comparison of two measures of similarity. [file 1471-2164-11-626-S11.DOC]
