# Supplementary material for: Genetic variation in the odorant receptors family 13 and the mhc loci influence mate selection in a multiple sclerosis dataset
Source: BMC Genomics. 2010 Nov 10;11:626. doi: 10.1186/1471-2164-11-626 (PMC3091764; doi:10.1186/1471-2164-11-626)
Supplement: Additional file 12 — Text S2. Comparison of two methods of assessing significance of Pearson Correlation as a measure of similarity. [file 1471-2164-11-626-S12.DOC]

Supplementary Figure -- Comparison of measures of similarity

A
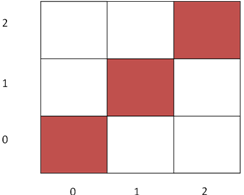
 B
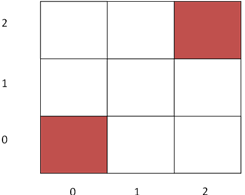
 C
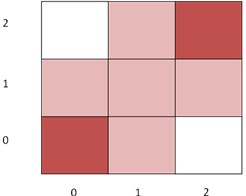


D
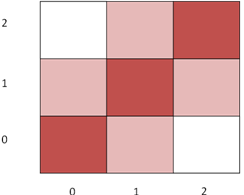
 E
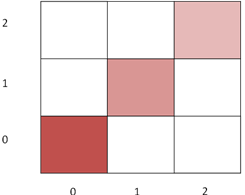
 F
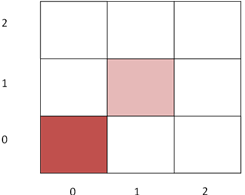


G
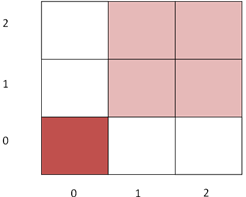
 H
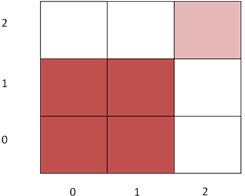


In these examples, for a given SNP, the genotypes of all females (0, 1, or 2) is plotted against the genotype of their spouses. Darker shades of red indicate greater frequency. Panels A-D highlight similarities between the R_chaix_ and R_pear_ measures, while panels E-H highlight key differences.

Panel (A) depicts a SNP for which all spouses have identical genotypes. Panel (B) shows a SNP for which there are no heterozygotes, and all spouses have identical genotypes (i.e. a female has a genotype of "2" if and only if her spouse has a genotype of "2"). Panel (C) indicates the type of situation that the R_chaix_ measure of similarity is designed to detect. Panel (D) shows a situation that the alternate measure of R_chaix_ (used in the Chaix et al paper but not in our paper) is designed to detect. Both R_chaix_ and R_pear_ are at a maximum in situation (B). The alternate R_chaix_ and R_pear_ are both at a maximum in situation (A).

Key differences between R_chaix_ and R_pear_ are highlighted in the remaining panels (E - H). In the case where the minor allele frequency is relatively low, say 5-15%, but spouses share identical SNPs (panels E and F), R_pear_ is at a maximum but R_chaix_ can be rather low. In the case where similarity at a SNP takes on a dominant model (i.e. a female has one or more copies of an allele if and only if her spouse has one or more copies of the allele) (G), R_pear_ can be quite high while R_chaix_ remains low. The same occurs when a recessive model is in play (H).

Note: The alternate R_chaix_, described in [1], is identical to the R_chaix_ used in this paper except that it assigns greater similarity to the case where both spouses are heterozygous (the difference between panel C and panel D).

1. Chaix R, Cao C, Donnelly P (2008) Is mate choice in humans MHC-dependent? PLoS Genet 4: e1000184.
